# Long-term survival of patients with pulmonary arterial hypertension recovering to World Health Organization functional class I or II: a historical comparison between intravenous epoprostenol and oral agents

**DOI:** 10.1186/1756-0500-7-359

**Published:** 2014-06-12

**Authors:** Koji Yamamoto, Yutaka Takeda, Yasuko Takeda, Taio Naniwa, Hitomi Narita, Nobuyuki Ohte

**Affiliations:** 1 Department of Cardio-Renal Medicine, Nagoya City University Graduate School of Medical Sciences, 1 Kawasumi, Mizuho-cho, Mizuho-ku, Nagoya, Aichi 467-8601, Japan; 2 Department of Cardiology, Nagoya City Rehabilitation Center, 1-2 Mikanyama, Yatomi-cho, Mizuho-ku, Nagoya, Aichi, Japan; 3 Department of Medical Oncology and Immunology, Nagoya City University Graduate School of Medical Sciences, 1 Kawasumi, Mizuho-cho, Mizuho-ku, Nagoya, Aichi, Japan

**Keywords:** Pulmonary hypertension, Survival, Epoprostenol, Endothelin receptor antagonist, Phosphodiesterase 5 inhibitor

## Abstract

**Background:**

Intravenous epoprostenol is the only drug proved in a randomized study to reduce mortality in patients with idiopathic pulmonary arterial hypertension (PAH). However, administration of this drug has procedural difficulties and a risk of sepsis. Oral drugs provide simple treatment, but their benefit for survival has not been proven. A recovery of patients with PAH to World Health Organization functional class (WHO-FC) I or II may predict favorable survival.

**Methods:**

Survival analyses were performed on a historical cohort of 41 patients with PAH. The patients were 43 ± 22 years old, 23 had idiopathic or heritable PAH, and 18 had connective tissue disease-associated PAH. The baseline was defined as the initial visit to a medical facility.

**Results:**

The median duration of follow-up was 1276 days (108 to 5389 days) and 21 patients died during this period. The estimated survival times for patients who received intravenous epoprostenol and did and did not recover to WHO-FC I or II were 4371 ± 577 days and 1172 ± 404 days, respectively. These times for patients who were not treated with intravenous epoprostenol and did and did not recover to WHO-FC I or II were 4717 ± 554 days and 925 ± 230 days, respectively. A Cox proportional hazard analysis gave a hazard ratio for death after recovery to WHO-FC I or II of 0.07 (P < 0.001). In contrast, use of intravenous epoprostenol was not a significant factor affecting survival (P = 0.96).

**Conclusions:**

Patients with PAH who achieve recovery to WHO-FC I or II without use of intravenous epoprostenol have similar survival to those who reach the same WHO-FC with use of intravenous epoprostenol. Benign survival of patients with PAH who have recovered to WHO-FC I or II may extend for several years after onset of the disease.

## Background

Pulmonary arterial hypertension (PAH) carries a significant risk of death and patients with PAH who respond poorly to drug therapy often die within a few years of onset [1,2]. Among the drugs for PAH, only intravenous epoprostenol has been proved to prolong survival, at least in idiopathic PAH, in a randomized trial [3-5]. However, intravenous epoprostenol has several associated difficulties, including the need for continuous administration via an infusion pump and a permanent tunneled catheter, and the possibility of serious adverse events including pump malfunction, local site infection, catheter obstruction, and sepsis [4,5]. In contrast, drugs such as endothelin receptor antagonists (ERAs) or phosphodiesterase type-5 inhibitors (PDE5Is) can be administered orally. However, despite numerous studies, the effects of ERAs and PDE5Is on survival have not been established [6-8] and meta-analyses have not proven a survival benefit for either drug class [6,7].

Based on this background and given the poor prognosis of PAH, patients and physicians often encounter a dilemma in selecting an appropriate treatment regimen. To address this difficulty, guidelines based on expert consensus on the diagnosis and treatment of PAH have set treatment goals that predict a benign course [4,5]. One goal is achievement of World Health Organization functional class (WHO-FC) I or II [4]. The WHO-FC is a powerful predictor of survival, despite large interobserver variation in the assessment [4,5,9,10], with median survival times of 6 months for WHO-FC IV, 2.5 years for WHO-FC III, and 6 years for WHO-FC I and II in untreated patients with idiopathic or heritable PAH [11]. Several other studies have reached similar conclusions [12-15].

Few studies have examined differences in survival after achieving WHO-FC I or II with different drugs. Intravenous epoprostenol is widely considered to be the most potent drug for PAH, whereas ERAs and PDE5Is are considered less effective [4,5]. However, it is unclear whether patients who achieve WHO-FC I or II with an ERA or PDE5I survive for as long as those reaching the same WHO-FC with epoprostenol. This information is important for choice of treatment with or without intravenous epoprostenol. Thus, the current study was performed to examine this issue.

## Methods

### Patients

**Figure 1:**
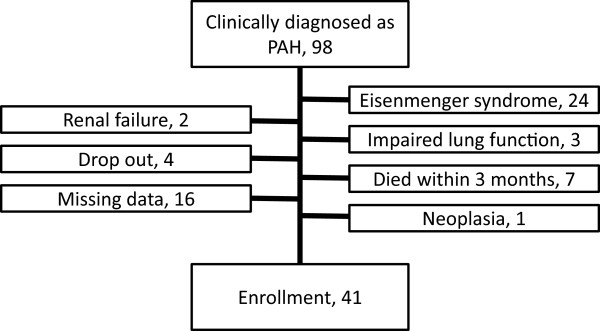
STARD flow chart of the disposition of patients in the study.

We retrospectively reviewed the medical charts of all patients with idiopathic or connective tissue disease (CTD)-associated PAH who were referred to our hospital between January 1, 2004 and March 31, 2012. PAH was defined using the standard definition: mean pulmonary arterial pressure ≥25 mmHg, pulmonary capillary wedge pressure ≤15 mmHg, and pulmonary vascular resistance >3 Wood Units [4,5]. We excluded patients with a diagnosis of pulmonary veno-occlusive disease or pulmonary capillary hemangiomatosis. This resulted in inclusion of 98 patients with PAH. We subsequently excluded patients with a congenital shunt because their survival clearly differs from patients with other types of PAH [16]. We also excluded patients with co-morbidities that could affect survival, such as those with malignancy. The 41 patients who were finally included in the study (Figure 1) were selected by a committee blinded to patient identification and clinical course after the beginning of management. The characteristics of the patients at the time of their initial visit to a medical facility were collected from medical records. The institutional ethics committee of our hospital approved the study protocol. The ethics guidelines of our institute, which is approved by the Ethics Committee of Nagoya City University Graduate School of Medical Sciences, do not require written informed consent from each patient on the condition that personally identifiable information is excluded from the study results.

### Survival analysis

We performed two survival analyses using different definitions of the baseline. In the first analysis, this was defined as the day of the first visit to a medical facility for a symptom of PAH. In the second analysis, the baseline was defined as the day of initiation of epoprostenol in patients administered this drug, and as the day of final escalation of medication in patients who did not receive intravenous epoprostenol. All patients were asked to visit our outpatient clinic every four weeks for adjustment of therapy and to allow collection of clinical information. In addition to these periodical contacts, the investigators and patients sometimes communicated by telephone. The patients were divided into 4 groups based on recovery or no recovery to WHO-FC I or II after 12-week treatment and treatment with or without intravenous epoprostenol: those who received intravenous epoprostenol and subsequently recovered to WHO-FC I or II (Group 1); those who received intravenous epoprostenol, but failed to recover to WHO-FC I or II (Group 2); those who did not receive intravenous epoprostenol, but recovered to WHO-FC I or II after treatment with another drug (Group 3); and those who did not receive intravenous epoprostenol and failed to recover to WHO-FC I or II (Group 4).

### Statistical analysis

Statistical analyses were performed with the Statistical Package for Social Science version 15.0 for Windows (SPSS Inc., Chicago, IL). Survival times were defined as the length of time a patient lived from the baseline date to Dec 31, 2012. Continuous variables except estimated survival time are expressed as the mean ± the standard deviation. For comparison among groups, chi-square test was used for categorical variables and one-way ANOVA followed by Bonferroni's post-hoc comparisons tests for continuous variables. The prognostic values of the variables were tested using Cox proportional-hazards regression analyses. The results are expressed as hazard ratios with 95% confidence intervals. A Kaplan-Meier estimate was used to calculate the average survival time and survival rate. Estimated survival times are expressed as the mean ± standard error (range of 95% confidence interval [CI]). A two-sided p value < 0.05 was considered statistically significant.

## Results

### Characteristics of patients

The characteristics of the patients at their initial visit to a medical facility are shown in Table 1. Most were in WHO-FC III (71%) and almost 25% were in WHO-FC IV. The numbers of patients with idiopathic or heritable PAH and CTD-associated PAH were similar. Hemodynamic data at first right heart catheterization are also shown in Table 1. These data reflect the advanced WHO-FC: the hemodynamic variables were fairly severe and the 6-minute-walk distances were relatively short. The 2 patients in WHO-FC II at the first visit to a medical facility both advanced to WHO-FC III and received further treatment: one with and one without intravenous epoprostenol. The duration of follow-up from the initial visit to a medical facility ranged from 108 to 5389 days and the 3^rd^ quartiles was 2769 days. Death occurred in 21 patients. Treatment at the end of follow-up (death or the end of the study) is shown in Table 2. More than half of the patients received epoprostenol with or without an ERA or a PDE5I. In Japan, epoprostenol, bosentan, ambrisentan, sildenafil, tadalafil, and beraprost are approved for treatment of PAH.

**Table 1:** Patient characteristics at the time of initial visit to a medical facility

**Variables**			**All**	**Group 1**	**Group 2**	**Group 3**	**Group 4**
Age (years) ^*^			43 ± 22	38 ± 18^‡^	48 ± 22	52 ± 21	66 ± 9^‡^
Women/Men^†^			34 (83) / 7 (17)	10 (24) / 0 (0)	6 (15) / 3 (7)	9 (22) / 0 (0)	9 (22) / 1 (2)
WHO-FC^†§^	II/III/IV		2(5) / 29(71) / 10(24)	0 (0) / 12 (29) / 1 (2)	0 (0) / 5 (12) / 4 (10)	2 (5) / 7 (17) / 0 (0)	0 (0) / 5 (12) / 5 (12)
Etiology^†^	Idiopathic		22(54)	9 (22)	6 (15)	2 (5)	5 (12)
	Heritable		1(2)	0 (0)	0 (0)	1 (2)	0 (0)
	CTD	SSc	12(29)	1 (2)	3 (7)	4 (10)	4 (10)
		MCTD	3(7)	2 (5)	0 (0)	1 (2)	0 (0)
		SLE	2(5)	1 (2)	0 (0)	1 (2)	0 (0)
		PM	1(2)	0 (0)	0 (0)	0 (0)	1 (2)
Hemodynamics^*^	mPAP (mmHg)		51 ± 16	57 ± 21	53 ± 5	43 ± 13	45 ± 11
	RAP (mmHg)		8 ± 6	6 ± 4^||^	8 ± 4	6 ± 4^#^	14 ± 8^||,#^
	CI (L/min/m^2^)		2.1 ± 0.6	2.1 ± 0.6	2.3 ± 0.5	1.9 ± 0.5	2.1 ± 0.6
	PVR (dyne/sec/cm^−5^)		1058 ± 492	1368 ± 439	981 ± 314	884 ± 526	1039 ± 605
	SvO_2_ (%)		60 ± 10	61 ± 9	50 ± 17	63 ± 9	60 ± 10
6MWD (m)^*^			196 ± 188	267 ± 164	150 ± 230	401 ± 39^**^	56 ± 106^**^

^*^Average ± standard deviation, ^†^Number (%) of patients, ^‡^p = 0.004 between Group 1 and 4, ^§^p = 0.010, ^**^p = 0.03 between Group 3 and 4, ^||^p = 0.02 between Group 1 and 4, ^#^p = 0.02 between Group 3 and 4 in RAP. CI: cardiac index, CTD: connective tissue disease, MCTD: mixed connective tissue disease, mPAP: mean pulmonary arterial pressure, PM: polymyositis, PVR: pulmonary vascular resistance, RAP: right atrial pressure, SLE: systemic lupus erythematosus, SSc: scleroderma, SvO2: oxygen saturation of mixed venous blood, WHO-FC: World Health Organization functional class, 6MWD: 6-minute-walk distance.

**Table 2:** Treatment at the end of follow-up (death or the end of the study)

**Treatment**	**Number (%) of patients**				
	**All**	**Group 1**	**Group 2**	**Group 3**	**Group 4**
Epoprostenol^*^	22 (54)	13 (32)	9 (22)	0 (0)	0 (0)
ERA^†^	9 (22)	1 (2)	0 (0)	4 (10)	4 (10)
PDE5I	14 (34)	3 (7)	1 (2)	4 (10)	6 (15)
Beraprost	3 (7)	0 (0)	0 (0)	1 (2)	2 (4)

Variables are number (%) of patients. ERA: endothelin receptor antagonist, PDE5I: phosphodiesterase 5 inhibitor. Some patients received more than one of the drugs. ^*^p < 0.001, ^†^p = 0.035.

The numbers of patients of Groups 1 to 4 (defined in the Methods) were 13, 9, 9, and 10, respectively. Comparisons among 4 groups found some statistically significant differences between Group 1 and 4 or Group 3 and 4 in age, WHO-FC, right atrial pressure, and 6-munte-walk distance (Table 1). ERA use was rarer in patients on epoprostenol than in those not (Table 2).

### Effects of recovery to WHO-FC I or II on survival period from the initial visit to a medical facility

**Figure 2:**
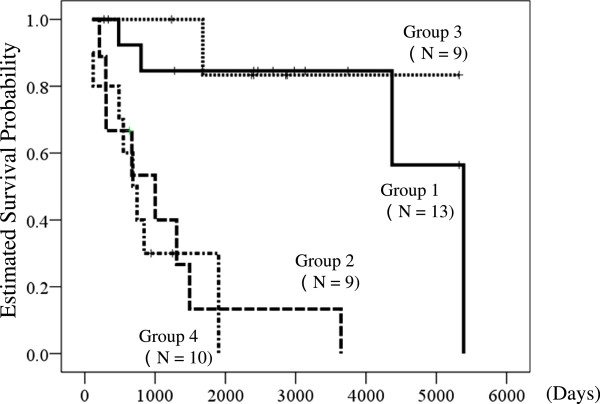
**Kaplan–Meier survival curves for patients from the day of their first visit to a medical facility.** The patients were divided into 4 groups: Group 1: intravenous epoprostenol and recovery to WHO-FC I or II; Group 2: intravenous epoprostenol, but failure to recover to WHO-FC I or II; Group 3: no intravenous epoprostenol, but recovery to WHO-FC I or II; and Group 4: no intravenous epoprostenol and failure to recover to WHO-FC I or II. Cox proportional hazard analysis gave a hazard ratio for death compared to recovery to WHO-FC II or better of 0.07 (95% confidence interval: 0.02 to 0.26, P < 0.001). Use of intravenous epoprostenol had no significant influence on survival (p = 0.96).

Kaplan-Meier estimates of survival from the first visit to a medical facility are shown in Figure 2. The survival curves for the 4 groups roughly divided into two types, with patients who recovered to WHO-FC I or II generally having longer survival than patients who did not achieve this recovery. Survival times in groups 1 to 4 were 4371 ± 577 days (3240 to 5503 days), 1172 ± 404 days (380 to 1695 days), 4717 ± 554 days (3630 to 5805 days), and 925 ± 230 days (474 to 1375 days), respectively. Cox proportional hazard analysis gave a hazard ratio for death of recovery to WHO-FC I or II of 0.07 (95% CI 0.02 to 0.26, P < 0.001). Intravenous epoprostenol had no significant influence on survival (P = 0.96).

### Effects of recovery to WHO-FC I or II on survival period based on medication initiation

**Figure 3:**
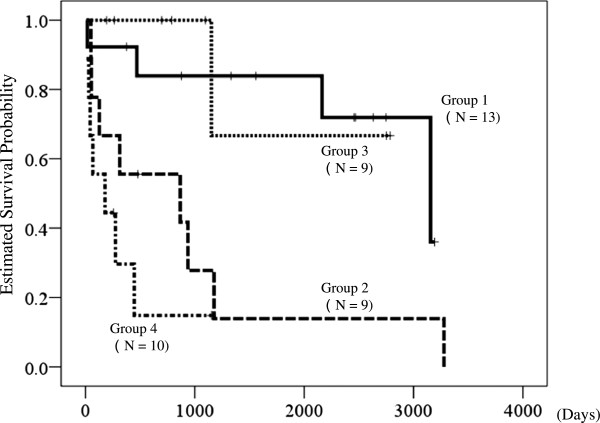
**Kaplan-Meier estimates of survival time from the start of intravenous epoprostenol or the last increase of oral medication.** The patients were divided into 4 groups: Group 1: intravenous epoprostenol and recovery to WHO-FC I or II; Group 2: intravenous epoprostenol, but failure to recover to WHO-FC I or II; Group 3: no intravenous epoprostenol, but recovery to WHO-FC I or II; and Group 4: no intravenous epoprostenol and failure to recover to WHO-FC I or II. Cox proportional hazard analysis gave a hazard ratio for death compared to recovery to WHO-FC II or better of 0.15 (95% confidence interval: 0.05 to 0.44, P < 0.001). Use of intravenous epoprostenol had no significant influence on survival (p = 0.33).

Kaplan-Meier estimates of survival from the start of intravenous epoprostenol or the last escalation of oral medication are shown in Figure 3. This analysis also resulted in each of the 4 groups falling into one of two types, with patients who recovered to WHO-FC I or II having longer survival than those who failed to show this recovery. Survival times in groups 1 to 4 were 2583 ± 311 days (1973 to 3192 days), 930 ± 390 days (165 to 1694 days), 2242 ± 445 days (1368 to 3114 days), and 314 ± 135 days (49 to 579 days), respectively. Cox proportional hazard analysis gave a hazard ratio for death of recovery to WHO-FC I or II of 0.15 (95% CI 0.05 to 0.44, P = 0.001). Again, intravenous epoprostenol had no significant influence on survival (P = 0.33).

### Recovery to WHO-FC I or II and survival in idiopathic and CTD-associated PAH

The numbers of cases of idiopathic or heritable PAH and CTD-associated PAH in groups 1 to 4 were shown in Table 1. The relationship between disease type and clinical course (groups 1 to 4) was not significant (P = 0.339). Cox proportional hazard analysis gave P = 0.174 for disease type and P = 0.001 for clinical course.

## Discussion

This study shows that recovery to WHO-FC I or II in patients with PAH increases the survival period, regardless of treatment with intravenous epoprostenol or an oral ERA or PDE5I. Among the patients, 25% were followed for more than 2750 days (7.5 years); thus, this is a follow-up study with a long observation period. Benign survival of patients who recovered to WHO-FC I or II seems to be preserved for at least 4000 days after their first visit to a medical facility. These results imply that meeting the treatment target is vital, but the specific drug therapy is less important in developing a treatment plan for a patient with PAH. This finding may assist patients and physicians in the choice of drug for PAH.

Guidelines recommend that the patient's status should remain in the range of the treatment goal, while few guidelines provide a comparison among drugs [4,5,17]. The European guidelines described the following drugs as recommendations for initial treatment in nonvasoreactive patients in WHO-FC III: ambrisentan, bosentan, sitaxentan (now withdrawn), sildenafil, intravenous epoprostenol, inhaled iloprost, tadarafil, and subcutaneous or inhaled treprostinil [4]. The expert consensus document from the United States recommends the following drugs for patients at lower risk, which fits with WHO-FC II or III: ERAs, PDE5Is, intravenous epoprostenol, intravenous or subcutaneous treprostinil, and inhaled iloprost [5]. Neither of these guidelines provide a reference for choice of one drug [4,5], probably because head-to-head comparisons of drugs are not available [4]. Thus, a particular drug cannot be selected in an evidence-based manner. The results of the current study provide confidence to patients and physicians that the selected drug is not very important if recovery to WHO-FC class I or II is obtained.

One of the weaknesses of the study is use of the WHO-FC as a marker of the degree of recovery, but not other medical parameters. The WHO-FC system was modified from the New York Heart Association functional class (NYHA-FC) [18] and both the WHO-FC and NYHA-FC depend on the physician’s interpretation of ordinary physical activity, slight limitation, or marked limitation [18]. This may cause high interobserver variability [10,19]. Despite this problem, the WHO-FC is a powerful predictor of survival and the severity of PAH is most often classified using the WHO-FC [4,5,12-15,20,21]. The WHO-FC has proved to be clinically useful and is used routinely in most randomized clinical trials. Improvement of WHO-FC after medical treatment predicts the long-term survival of patients with PAH [12,15], with patients in WHO-FC I or II at baseline and follow-up after drug therapy having a survival probability of 90% after 5 years, while those in WHO-FC III or IV before and after drug therapy have a survival probability of 34% after 5 years [15]. Patients with PAH who improve from WHO-FC III to I or II after drug therapy also have better survival than those who remain in WHO-FC III [12,15]. Thus, despite the large interobserver variability, the WHO-FC is valuable for assessment of the status of patients with PAH. This indicates that the validity of the results in the current study is decreased little by the lack of other clinical parameters.

Group 4, a group of patients who did not receive intravenous epoprostenol and failed to recover to WHO-FC I or II, showed some particular characteristics (Table 1). Intergroup comparison found that their ages were older and their right atrial pressure and 6-minute-walk distance were worse than those of patients who recovered to WHO-FC I or II (Group 1 or 3). This suggests that some of them gave up intravenous epoprostenol because of their advanced age and inability of self-supervision of home therapy, and that some of them did so due to too advanced disease.

This study has several other limitations. First, it was a retrospective study. Second, as a single-center study, some referral bias may be present. Third, the limited sample size may impair the statistical power of the analyses. Fourth, comparisons between ERAs and PDE5Is and between idiopathic or heritable PAH and CTD-associated PAH were not performed because of the limited sample size. Fifth, this study did not consider changes in treatment after the first 12 weeks following initiation of epoprostenol (in patients administered this drug) or final escalation of medication (in patients who did not receive intravenous epoprostenol). Within these limitations, the results of the study suggest that patients who recover to WHO-FC I or II have a better prognosis than those without this recovery, regardless of the disease type and drug treatment. Validation of this finding will require a trial in a larger population.

## Conclusion

Patients with PAH who achieve recovery to WHO-FC I or II with ERAs or PDE5Is survive for as long as those who reach the same WHO-FC with intravenous epoprostenol. Benign survival of patients who recover to WHO-FC I or II may extend for several years after onset of the disease.

## Abbreviations

BNP: Brain natriuretic peptide; CI: Confidence interval; CTD: Connective tissue disease; ERA: Endothelin receptor antagonist; NYHA-FC: New York Heart Association functional class; PAH: Pulmonary arterial hypertension; PDE5I: Phosphodiesterase type-5 inhibitor; WHO-FC: World Health Organization functional class.

## Competing interests

The authors declare that they have no competing interests.

## Authors’ contributions

KY is the main writer of Abstract, Methods, Results, Tables and Figures, and collected data of patients. YT (Yutaka) conceived of and designed the study, and is the main writer of Background and Discussion. YT (Yasuko), TN, HN and NO are the committee for assessment of eligibility of each patient. NO helped to draft the manuscript. All authors read and approved the final version of the manuscript.
